# Using a recreational grade echosounder to quantify the potential prey field of coastal predators

**DOI:** 10.1371/journal.pone.0217013

**Published:** 2019-05-22

**Authors:** Tom Brough, William Rayment, Steve Dawson

**Affiliations:** Department of Marine Science, University of Otago, Dunedin, New Zealand; University of Waikato, NEW ZEALAND

## Abstract

Quantifying the distribution of prey greatly improves models of habitat use by marine predators and can assist in determining threats to both predators and prey. Small epipelagic fishes are important prey for many predators yet their distribution is difficult to quantify due to extreme patchiness. This study explores the use of recreational grade echosounders (RGE) to quantify school characteristics of epipelagic fish and link their distribution to that of their predators at Banks Peninsula, New Zealand. The hydro-acoustic system was ground-truthed with 259 schools of epipelagic fish. During 2015 and 2016, 136 hydro-acoustic surveys were conducted with concurrent observations of Hector’s dolphin (*Cephalorhynchus hectori*) and little penguins (*Eudyptula minor*). The relative abundance of the two predator species during surveys was modelled according to the relative abundance of potential prey using generalised additive mixed models. Schools of epipelagic fish were readily detected by the RGE system and were more abundant in summer compared to winter. The models performed well, explaining 43% and 37% of the deviance in relative abundances of dolphins and penguins respectively. This is the first study to link the distribution of Hector’s dolphin to that of their epipelagic prey and confirms the utility of RGE in studies of habitat use in marine predators. Limitations associated with a lack of formal acoustic calibration and data formatting can be overcome and would make RGE valuable, inexpensive tools for investigating variability in populations of small pelagic fishes.

## Introduction

The distribution of marine top predators generally reflects that of their prey [1–3]. For this reason, studies investigating habitat use of predators greatly benefit from data that quantify prey [4–6]. Such data have been shown to improve the predictive power of habitat models [7,8], elucidate threats associated with prey depletion [9,10], and can contribute to marine spatial planning [11,12].

Patchiness over multiple temporal and spatial scales [2,5,13], is the main challenge with sampling the pelagic prey that make up the diet of a wide range of marine predators [10,14,15]. Increasingly, hydro-acoustics are used to obtain data on small epipelagic fishes [14,16,17]. These methods offer many advantages for quantifying prey fields including ability to integrate prey data over multiple spatiotemporal scales [18,19], capacity to measure the patch characteristics of prey [5,15], and the compatibility of the method with concurrent observations of predators [14,18,20]. In addition, there are clear advantages in the method being non-destructive.

For many research programmes the significant cost involved with the purchase or hire of a scientific echo-sounder (SES), and the expertise or logistic requirements to operate such equipment, present major hurdles. These factors may compromise the repeatability of surveys and therefore constrain the sample size required to resolve a patchy prey field. Several modern recreational grade echo-sounders (RGE) allow on-board recording of the digital acoustic data, and could, within certain limitations, provide an alternative to SES. RGEs have been used to quantify aspects of the prey community in deep water habitats [20] and coastal settings [17,21], as well as for mapping fish schools over shallow coral reefs [22]. However, lack of information on the basic operational parameters required to format (and subsequently calibrate) backscattered energy in a way that allows hydro-acoustical analysis (i.e. as scattering volume (Sv)), compromise the ability of RGE to quantify the abundance of prey. Without calibration or reference to a calibrated system [21], RGE systems can only quantify ‘potential prey’ in a relative sense. Yet, given the generalist diet of many predators and their preference for prey taxa that are most abundant [20,23], this may not be a significant drawback.

Little is known about the distribution of epipelagic schooling fish in New Zealand waters, particularly at the fine scales relevant to determine overlap with marine predators, see [20,24–26] for the few exceptions. Demonstrated impacts on epipelagic fish communities from climate change [27,28] and overfishing [9,29] add further weight to the need to understand the spatial ecology of these important taxa.

**Fig 1:**
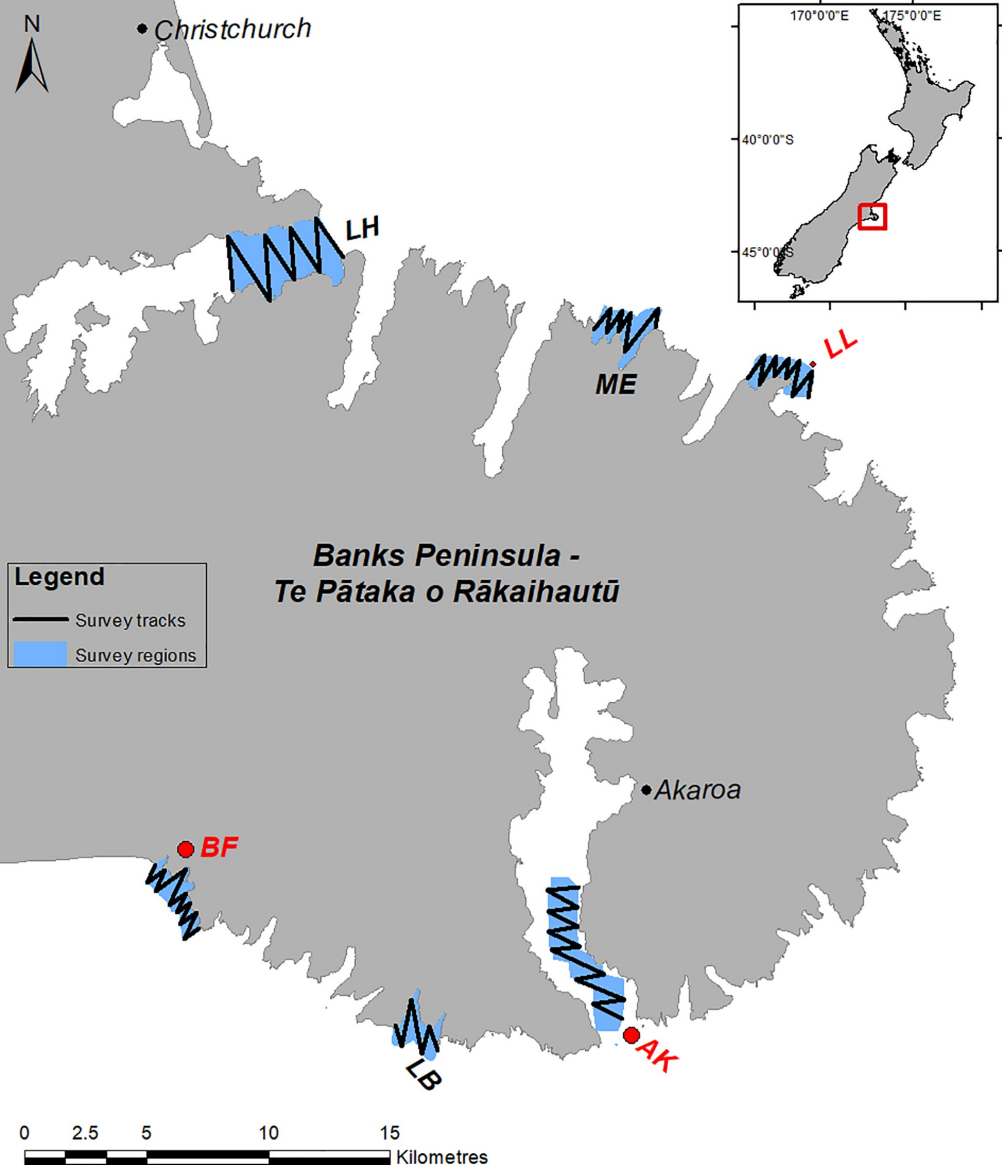
The six survey regions at Banks Peninsula, New Zealand. BF is Birdling’s Flat, LB is Long Bay, AK is Akaroa Harbour, LL is Long Lookout, ME is Menzies Bay and LH is Lyttelton Harbour. The three hotspots for Hector's dolphins are shown in red font. Example survey tracks for the predator-prey surveys are shown at two survey regions. Inset shows the location of Banks Peninsula on the New Zealand coastline.

Banks Peninsula (-43.8; 173.1.E), on the east coast of New Zealand’s South Island (Fig 1), has an abundance of marine predators that have been shown to target epipelagic prey [23,30]. Hector’s dolphin (*Cephalorhynchus hectori*) is an endangered coastal dolphin that is found in shallow (<100m) waters around the South Island of New Zealand. Banks Peninsula is a stronghold for this endemic species. The dolphins have a generalist diet focussing on species throughout the water column, but epipelagic prey (e.g. sprat, pilchard and mullet) contribute significantly to their diet [30]. Both little penguins (*Eudytptula minor)* and an endemic and endangered subspecies, white-flippered penguin (*Eudytptula minor albosignata)*, co-exist at Banks Peninsula; hereafter little penguin refers to both subspecies. The penguins are central-place foragers, making foraging trips (usually daily) within 20km from fixed nesting colonies [31–33]. A dominant component of their diet is small epipelagic, clupeiform fish such as pilchard, anchovy and sprat [23,34]. Substantial declines in populations of little penguin have been linked to fluctuations in the abundance of these important epipelagic taxa [32,35,36]. No formal studies of epipelagic species have been undertaken at Banks Peninsula but species such as slender sprat (*Sprattus antipodum)* are known to be particularly abundant in waters around the peninsula [37,38]. Yellow-eyed mullet (*Aldrichetta forsteri*) are also common throughout coastal waters of NZ [39] and are seasonally abundant at Banks Peninsula. Pilchard (*Sardinops neopilchardus*), though generally more abundant in warmer, northern NZ, has also been recorded in large numbers in the south [40] and anecdotally seem to be common at Banks Peninsula in the summer. Together, sprat, mullet and pilchard are the major species of epipelagic fish at Banks Peninsula. These species form large aggregations in the nearshore habitat (pers. obs.), and should be readily detected by RGEs. These features provide an opportunity to trial the use of an RGE to quantify aspects of the epipelagic fish community, and relate these to the distribution of predators.

## Materials and methods

As all field work for this study was carried out within NZ territorial seas, no specific permissions were required from any agency. All field research on the endangered Hector’s dolphin was carried out in accordance with the New Zealand Marine Mammal protection act 1978. No collection of wild animals or animal parts was undertaken.

### Hydro-acoustic systems

The hydro-acoustic systems used in this study were two similar ‘off the shelf’ RGE produced by Lowrance Marine Electronics (Tulsa, USA) and Simrad (Simrad Ltd. Oslo, Norway). The Lowrance was a 2014 Elite-7 that powered a hybrid dual imaging (HDI), multi-frequency, dual beam transducer with two elements capable of transmitting and receiving at 50 or 200kHz and 455 or 800kHz. The transducer was hull-mounted 0.5m below the waterline on the transom of an outboard driven 6m aluminium hulled research vessel. The Simrad system (2016 NSS7 Evo2) used the same transducer.

Both systems offered some user control of operational settings. Ping rate and gain were set manually after field trials to find optimum values for the study area (Table 1). Source level is automatically configured to the various range settings and could not be quantified reliably or set manually. For these surveys, the systems were set to ‘shallow water mode’, which sets pulse width at 0.2ms and applies an unknown time varied gain (TVG) function to water column samples (Navico pers. comm.). Both Simrad and Lowrance are owned and operated by the same parent company (Navico Ltd, Lysake, Norway). Consequently the two echo-sounders were very similar in their operation and, importantly, in the way they stored acoustic data. Navico echo-sounders store data on raw echo returns written to a compressed format in a ‘.sl2’ file. The files consist of binary strings that code for particular parameters associated with the echo return and navigation. Both units also have in-built GPS receivers so latitude, longitude and precise UTC time data are stored in the GPS string respective for every ping.

**Table 1:** Echosounder settings.

Parameter	Simrad NSS7 evo2	Lowrance Elite-7
Transducer	HDI 50/200 455/800 kHz	HDI 50/200 455/800 kHz
Max depth	755 m (@ 50 kHz)	755 m (@ 50 kHz)
3dB beam angle	12^o^	12^o^
Frequency	200 kHz	200 kHz
Ping rate	9–13 Hz	9–13 Hz
Sampling rate	3 MHz	1 MHz
Pulse width	0.2ms	0.2ms
Gain	System value: 5	System value: 55
Time Varying Gain (TVG)	None	None
Output power	1000 W RMS (Max)	250W RMS (Max)
Source level	Range specific/unknown	Range specific/unknown

Relevant operational settings for both hydro-acoustic systems used in this study. Both systems used the same transducer.

### Ground-truthing

**Fig 2:**
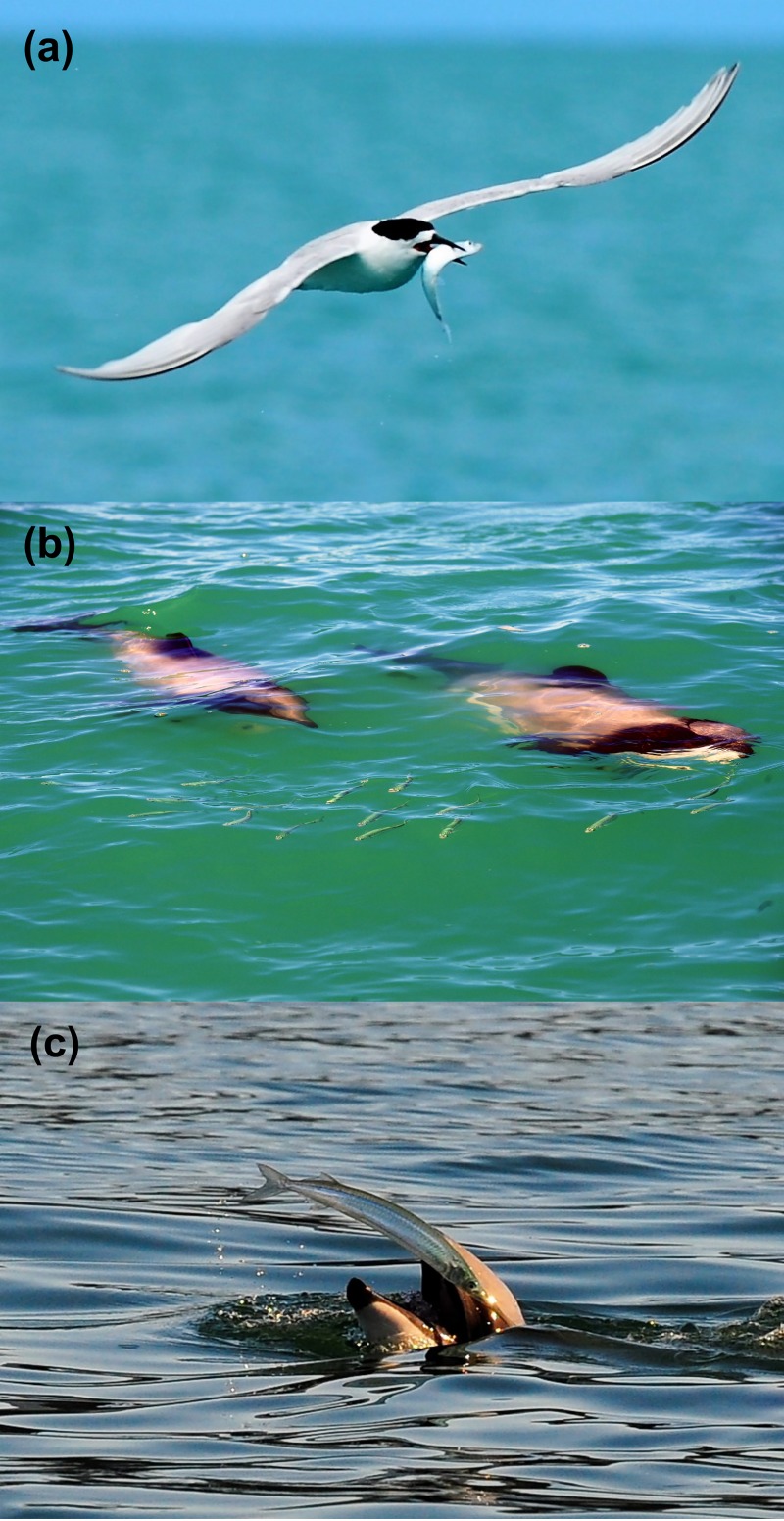
Photographic examples of prey identification from ground-truthing events. The three most commonly encountered prey species are shown; (a) a slender sprat captured by a white fronted tern, (b) Hector's dolphins corralling a school of NZ pilchard and (c) a yellow-eyed mullet being caught by a Hector’s dolphin.

While RGE systems have well-documented capabilities for detecting and recording fish schools [17,21,22], some background information is required to classify echogram marks as schools. Information typically used to identify echogram marks [16,26,41], such as fish behaviour, school dimensions, and/or target strength relationships, is unavailable for Banks Peninsula. Therefore to aid in the discrimination of fish schools in the acoustic data gathered in the systematic predator-prey surveys (see below), we ground truthed the hydro-acoustic system with known epipelagic schools at Banks Peninsula between 2015 and 2017.Ground-truthing was used to provide information on school morphology and relative scattering strength of epipelagic fish schools in our study area that were clearly relevant for coastal predators. Such information was important to; 1) establish appropriate minimum school dimensions in the algorithms used to detect schools during systematic surveys, and 2) ensure that the minimum threshold value for the relative intensity values did not degrade schools of potential prey and 3) provide information on relative scattering strength of prey schools. Data on relative scattering strength can help to distinguish prey schools from other echogram marks (e.g. sediment plumes, zooplankton). During ground-truthing, fish schools were located opportunistically by visually identifying aggregations at the surface or, more commonly, observing predators corralling and actively foraging on epipelagic species. A ground-truthing ‘event’ was an instance in which epipelagic aggregations were confirmed, were stable for at least 5 minutes prior to hydro-acoustic data logging, in good weather (Beaufort sea state <3, swell <1.5m). Hydro-acoustic and navigation data were logged continuously during each ground-truthing event, with the vessel manoeuvring to ensonify a volume of water as close as possible to where schools had been observed. Georeferenced notes, entered into a HP-palmtop computer connected via serial port to the GPS chartplotter, included information on the top predators present, fish species (if possible), weather conditions, survey speeds and directions. When possible we used a Nikon D3 DSLR camera with an 80-200mm f2.8 zoom lens to photograph foraging predators and confirm the species being preyed upon (Fig 2). We could not identify species for all schools observed during ground-truthing. Identifying the composition of schools in ground-truthing events was done solely to establish which species were most likely to constitute the epipelagic prey field and to match this information to studies of diet of the predators from the study area. Many diving predators (especially spotted shags, *Phalacrocorax punctatus*), surfaced with prey before consuming them. In this way we were able to identify some schools that were deeper than we could visually observe. Ground-truthing events were separate from the systematic surveys used to investigate the overlap between predators and their prey (see below). Schools detected in the systematic surveys were not ground truthed and hence are described as ‘potential’ prey because their species composition was unknown.

Hydro-acoustic and navigation data were written to a micro-SD card in .sl2 file format. These files were read using the software Sonar TRX (Leerand Engineering Inc.), and the raw data exported as comma separated values (.csv). Data were formatted for analysis using *R* (version 1.0.153; R Core Team 2017). Formatting steps included: 1) Converting UTC timestamps into NZ standard time, 2) Selecting the required variables from the dataset (i.e. date, time (to the nearest millisecond), latitude, longitude, ping number, sampling rate, max/min range, total number of samples per ping and the full sample count, and 3) Transforming the sample count from a linear 8-bit integer to dB scale. This was undertaken with information supplied by the manufacturer. However, due to the proprietary nature of the material we were not able to obtain information concerning the acoustic source levels or gain functions. Without these parameters, it was not possible to map the data to scattering volume (Sv;[42]) format (i.e. the typical form used for calculating estimates of abundance and density in hydro-acoustical analysis; [43]. However, the dimensions of schools can be defined from raw–dB acoustic backscatter data [22,43], and thus alternative measures of epipelagic relative abundance were calculated (see below).

Hydro-acoustic and navigation data for each ground-truthing event were imported into the software Echoview version 7.1 (Echoview Software Pty Ltd) for analysis. There was limited information available on the application of time variable gain (TVG) to the data stored by our RGE. Thus, it was possible that backscatter data sourced from the RGEs were depth dependent. To quantify and correct the correlation between the relative intensity values and depth, we recorded the intensity returns of a 38.1mm tungsten carbide calibration sphere, lowered directly below the transducer. On-axis samples were recorded between the depths of 3 and 35m (the maximum depth of the study area). Water column samples that contained acoustic backscatter originating from the calibration sphere were isolated in Echoview in Sv format by constructing 8 x 1 (vertical by horizontal) regions around the perceived position of the sphere. Mean relative Sv values were generated for each region. Plotting the correlation between mean relative intensity values of the sphere and depth provides an indication of any TVG applied to the data and allows the application of additional TVG curves, should the data require it. Further insights into utility of the TVG correction were given by plotting the relationship between mean relative Sv values and depth for schools detected during ground-truthing, before and after additional TVG application. The process to assess and remove depth dependence of acoustic backscatter was repeated with each of the two echo-sounders used in this study, with the settings that were used during ground-truthing and predator-prey surveys. The final TVG form (i.e. 40*log*, 20*log*, 15*log* or 10*log)* was decided by the form that minimised correlation (positive or negative) between relative intensity and depth. The TVG function is:

Y=ξlog(R)+2αR

Where Y is the TVG function at range (R), ξ is the TVG range coefficient that is set to 10 for cylindrical spreading and α is the acoustic absorption coefficient. *Y* is applied to the raw data to remove the depth dependency of the intensity values. The frequency specific acoustic absorption is defined as:

$$\alpha =10\;{log}_{10}[I(z)/I(z+\Delta z)]/\Delta z$$

Where *I* is the intensity of a backscattered wave, and z is the depth below the transducer given by a cartesian coordinate system [42]. Absorption is calculated for a particular speed of sound [43], thus temperature and salinity data were sampled using an RBR Concerto CTD (RBR Ltd, Ottawa, Canada), pH was set at an appropriate value (8) for sea water in this region and transmit frequency set at 200kHz. These values were used to formulate *α* using inbuilt functions in Echoview.

The analysis domain for school detection was set using Echoview’s ‘best candidate’ bottom picking algorithm fitted to the acoustic data to remove the seafloor, and an editable line was fixed at 3m to remove the acoustic near-field. A background noise removal function was then used to remove any unwanted noise from the echogram; generally a product of the signal to noise ratio decreasing with range.[44]. To detect schools of potential prey, the Shoal Analysis And Patch Estimation System (SHAPES) algorithm [45] was applied in Echoview. Horizontal resolution varied according to ping rate and vessel speed, but was generally between 20 and 34 cm. Vertical resolution was approximately 15 cm. Minimum analysis threshold was set at -35 dB relative intensity. This value effectively removed low intensity scattering sources whilst keeping the integrity of detected schools of potential prey. SHAPES was applied with conservative constraints on minimum school dimensions (1.5m thickness and 3m length). Echograms with detected schools were visually screened to ensure acoustic signals from surface noise, bubbles or wake were not included. The dimensions, depth range, and relative mean intensity (Echoview’s mean Sv) of schools were exported and plotted, providing a frequency distribution of school dimensions and school backscatter values for potential prey schools that were clearly relevant for the top predators in this location.

For this study, the parameters of interest produced by SHAPES included uncorrected length (*L*), uncorrected thickness *(T*) and uncorrected area (*A*). These parameters were then corrected for beam geometry following [46]such that corrected length (Lc) is:

Lc=L−(2×D×tan(ϕ/2)

Corrected thickness (Tc) is:

Tc=T−C/2×τ/1000

Corrected school area (Ac) is:

$$Ac=A\times \frac{\left(Lc\times Tc\right)}{\left(L\times T\right)}if\;L\times T\neq 0$$

Where D is mean school depth, ϕ is the 3dB beam angle, C is the speed of sound, and τ is the transmitted pulse length.

### Predator-prey surveys

Systematic surveys were carried out in nearshore habitat (<1km from shore) at Banks Peninsula in order to link the distribution of predators to that of potential prey. Surveys were undertaken in six survey regions (Fig 1) around the peninsula in summer (Jan-March) and winter (Aug-Oct) in 2015 and 2016. Water depth at the survey regions ranged from 8 to 35m. Three regions were known hotspots for Hector’s dolphin, with the other three being randomly selected ‘reference areas’(see [47] for details). Surveys followed a ‘zig-zag’ pattern in an alongshore direction (Fig 1), at survey speeds between 5 and 6 knots. Counts of Hector’s dolphins and little penguins were made by two observers concurrently with hydro-acoustic data acquisition. These species were selected because they have been demonstrated to use the epipelagic prey field [23,30], are common within the study area, and represent two very different taxa.

Hydro-acoustic data from each predator-prey survey were formatted as above. Schools of potential prey were detected by SHAPES and used minimum dimensions based on schools detected during ground-truthing events (5m length and 3m thickness). The relative abundance of potential prey (RAPP) for a given survey was calculated to provide a ‘snapshot’ of prey availability at a survey region and summarised as two metrics based on the dimensions of potential prey schools. The cumulative school area (c.SchA) was the summation of the area occupied by all schools detected in a survey, standardised by survey distance (m^2^/km). RAPP was also summarised as the proportion of a survey track over which schools were detected ([43] Prop.Track) by summing Lc over all detected schools, and dividing it by the survey distance. Generalised additive mixed models (GAMMs) were used to model the relationship between RAPP and predator counts in package *mgcv* [48] in *R*. The count of each predator species was used as the response variable in separate model families. Models were fitted using a negative binomial distribution and log-link function. The influence of unequal survey effort among surveys was accounted for by incorporating survey distance e.g. [49,50]. To determine the best RAPP metric for each predator, two separate models were fitted with either c.SchA or Prop.Track as predictors. The predictors were fitted with cubic splines with the number of degrees of freedom for each smoothed term being determined by generalised cross validation [51] with a maximum of 4 knots to prevent over-smoothing. Each model included a random effect term of survey region to account for autocorrelation among surveys from the same region. The best RAPP metric for each predator was determined by the model with the lowest AIC score [52]. The utility of the RGE data for defining predator-prey overlap was appraised by the performance (in terms of deviance explained) of the best metric for each predator, and the magnitude of the effects evident in plots of the smoothed terms. Model assumptions (i.e. independence, homogeneity of variance, under-smoothing) were checked using standard model diagnostic approaches [51,53].

## Results

Thirty-six ground-truthing events were carried out on observed schools of epipelagic fish. The majority of these (94%) occurred during summer when foraging aggregations are more common in the study area. Schools were detected acoustically in 86% of ground-truthing events. Prey species identification was possible either visually or photographically in 55% of ground-truthing events; in the remaining cases prey were either not seen sufficiently clearly, or were unknown species (possibly juveniles). The most common prey species observed were the slender sprat, followed by NZ pilchard (*Sardinops neopilchardus)* and yellow-eyed mullet (*Aldrichetta forsteri*) (Fig 2). Each of these species feature prominently in the diet of Hector’s dolphins and little penguins [23,30]. The most common predators associated with foraging events were white-fronted terns (*Sterna striata)*, Hector’s dolphins and spotted shags. Other taxa often encountered during ground-truthing events included predatory fish such as barracouta (*Thyrsites atun*) and kahawai (*Arripis trutta)* and the juvenile squat lobster (*Munida gregaria*). Acoustic signals of *Munida* were similar to prey schools identified as fish but were typically higher intensity, shallower and had much larger dimensions. The large number of ground-truthing events that contained ‘unknown’ epipelagic prey as well as the opportunistic nature of ground-truthing limits analysis of the locations or school morphology of the different epipelagic species.

**Fig 3:**
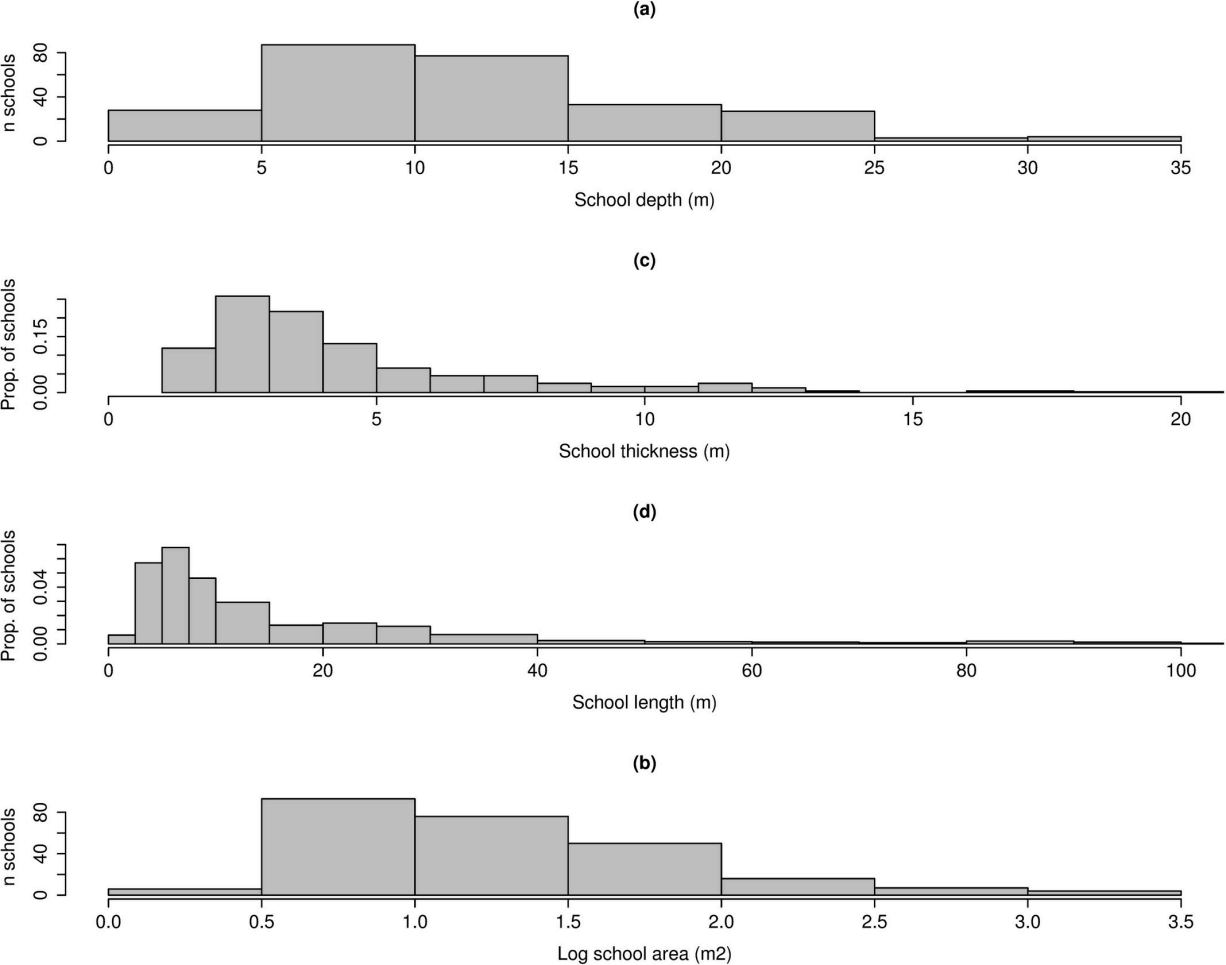
Histograms of the distribution of school dimensions. Dimensions are sourced from all schools detected in ground-truthing events. The mean depth of schools is given in (a), (b) is the distribution of school area, (c) school vertical thickness and (d) is school length.

Two hundred and fifty nine schools were classified as potential prey during ground-truthing. These showed wide variety in mean depth, ranging from 3 to 34m (Fig 3). School area was similarly variable with the majority of schools being between 5 and 100m^2^ in area. School thickness was strongly clustered at values less than 10m with a peak between 2 and 5m. Similarly, the highest proportion of school lengths was <20m, although schools up to 100m in length were observed. Although many small clusters of samples were detected, 84% of classified schools had dimensions greater than 2m vertical thickness and 5m length (Fig 3). Thus, minimum school dimensions of 2m thickness and 5m length were used in the SHAPES algorithm for data from the predator-prey surveys.

**Fig 4:**
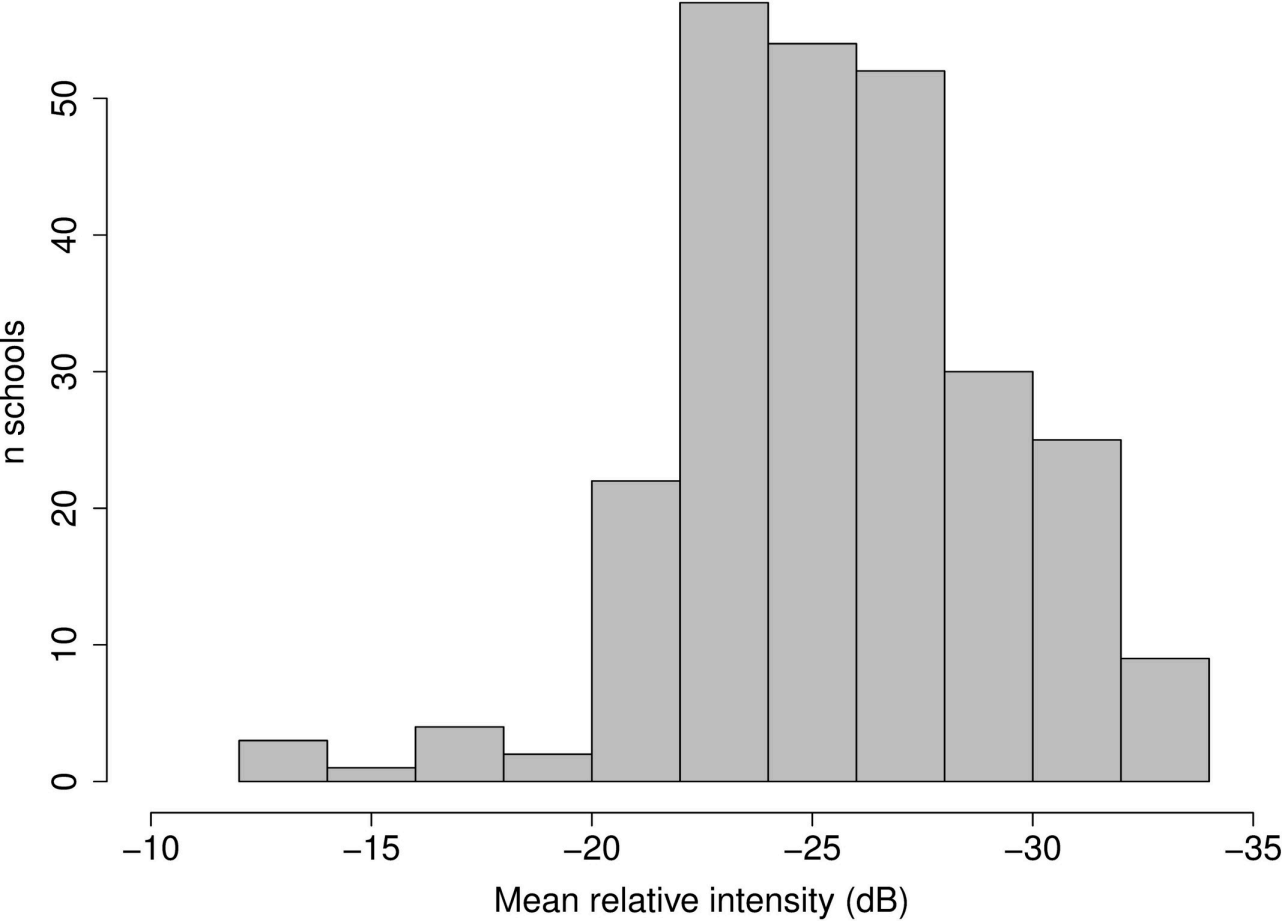
Distribution of mean intensity for schools of potential prey. Schools were detected during ground-truthing events. As the hydro-acoustic systems used in this study are not calibrated and there is limited information on crucial parameters concerning the echo-sounders’ transmit and receive functions, the data represent relative intensity only. Data have been corrected for depth dependence by the application of a TVG function.

The relative mean intensity of potential prey schools detected in ground-truthing ranged between -34 and -13 dB (Fig 4). The peak in the distribution of mean school intensity was -26 dB. The majority of detected schools had mean intensity values between -30 and -20 dB of relative intensity. Very few schools had high mean intensity above -20 dB (Fig 4).

**Fig 5:**
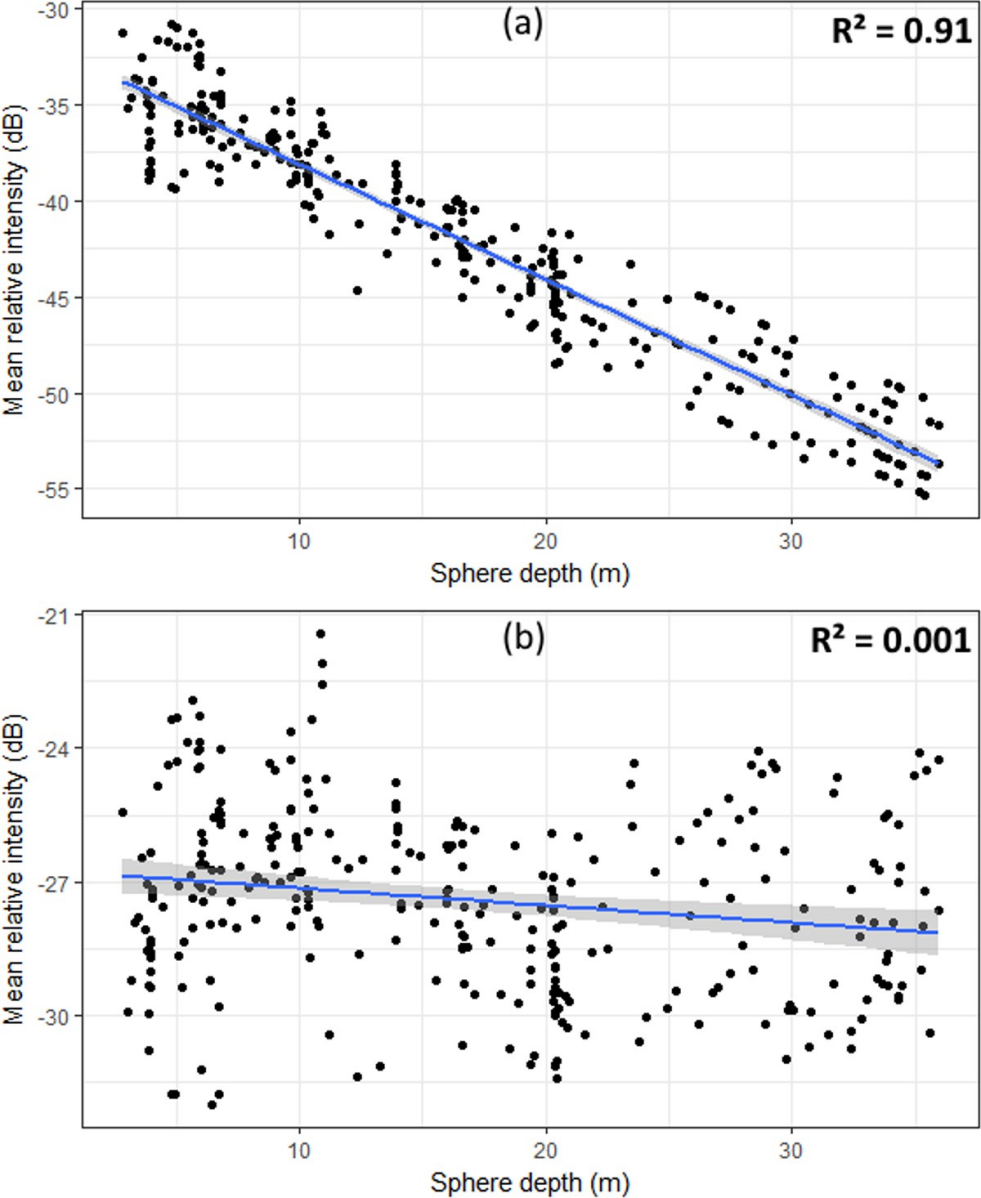
Calibration sphere. Plot of mean relative intensity of acoustic backscatter from the calibration sphere before (a) and after (b) the application of a 10 log TVG curve to remove the depth dependence of the data.

Three hundred regions of acoustic backscatter originating from the calibration sphere were isolated for each RGE. Acoustic relative intensity were clearly depth dependent as evident from viewing the echo returns from the calibration sphere and plotting the mean relative Sv of detected schools against depth. Application of the 20log function (the nominal TVG form for Sv data [54],) clearly overcompensated for the transmission loss, as did 40log (the function typically used in storing target strength (TS) data). The TVG form that best minimized the correlation between relative intensity and depth for these RGE was 10log, which removed the relationship between the intensity values of the calibration sphere and depth (Fig 5). Further, application of a 10log TVG curve removed the correlation between the mean Sv of prey schools and depth (S1 Fig). Removal of this correlation ensures that the threshold value applied to the hydro-acoustic data does not introduce bias to measurements of schools at certain depths (e.g. by degrading school boundaries) and means that school relative scattering strength is comparable among depth strata.

**Fig 6:**
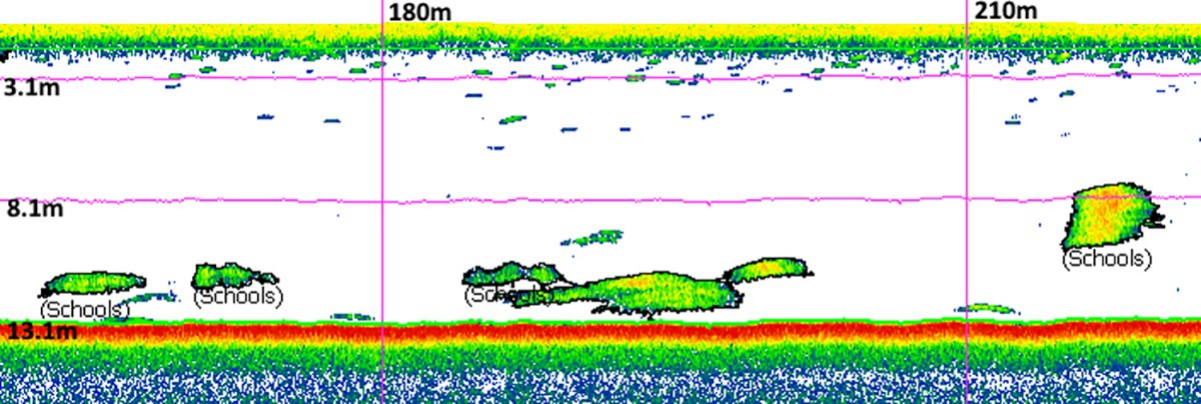
Echogram of hydro-acoustic data from a recreational grade echosounder. This echogram was obtained during predator-prey surveys. Schools of potential prey detected by SHAPES in Echoview are shown. The grid represents distance along-track (x dimension) and depth below the transducer (y dimension).

One hundred and thirty six predator-prey surveys were conducted over the six survey regions. Schools were readily detected by the RGE during these surveys (Fig 6). Surveys were not apportioned equally among regions due to variable weather and remoteness limiting sampling at some locations. There were similar numbers of surveys in winter and summer for each region. Predator counts were highly variable among regions; with dolphins being most abundant at Birdling’s Flat, Akaroa and Long Lookout. Penguin counts were highest at Akaroa, Birdling’s Flat and Long Bay (Table 2).

**Table 2:** Summary of surveys.

*Region*	μ *Dolphin* ± se	*μ Penguin* ± se	*Summer surveys*	*Winter surveys*	*Total surveys*
***AK***	5.92 ± 1.33	2.35 ± 0.52	19	20	39
***BF***	10.83 ± 2.39	0.78 ± 0.31	10	8	18
***LB***	2.93 ± 1.43	0.73 ± 0.25	9	6	15
***LL***	6.38 ± 1.31	0.23 ± 0.12	12	14	26
***LY***	0.86 ± 0.36	0.07 ± 0.06	7	8	15
***ME***	1.82 ± 0.82	0.57 ± 0.15	10	13	23
			67	69	136

Mean counts of two predators (Hector’s dolphin and little penguin) among survey regions, and the distribution of predator-prey surveys among seasons and survey regions in this study. Region codes are given in Fig 1.

**Fig 7:**
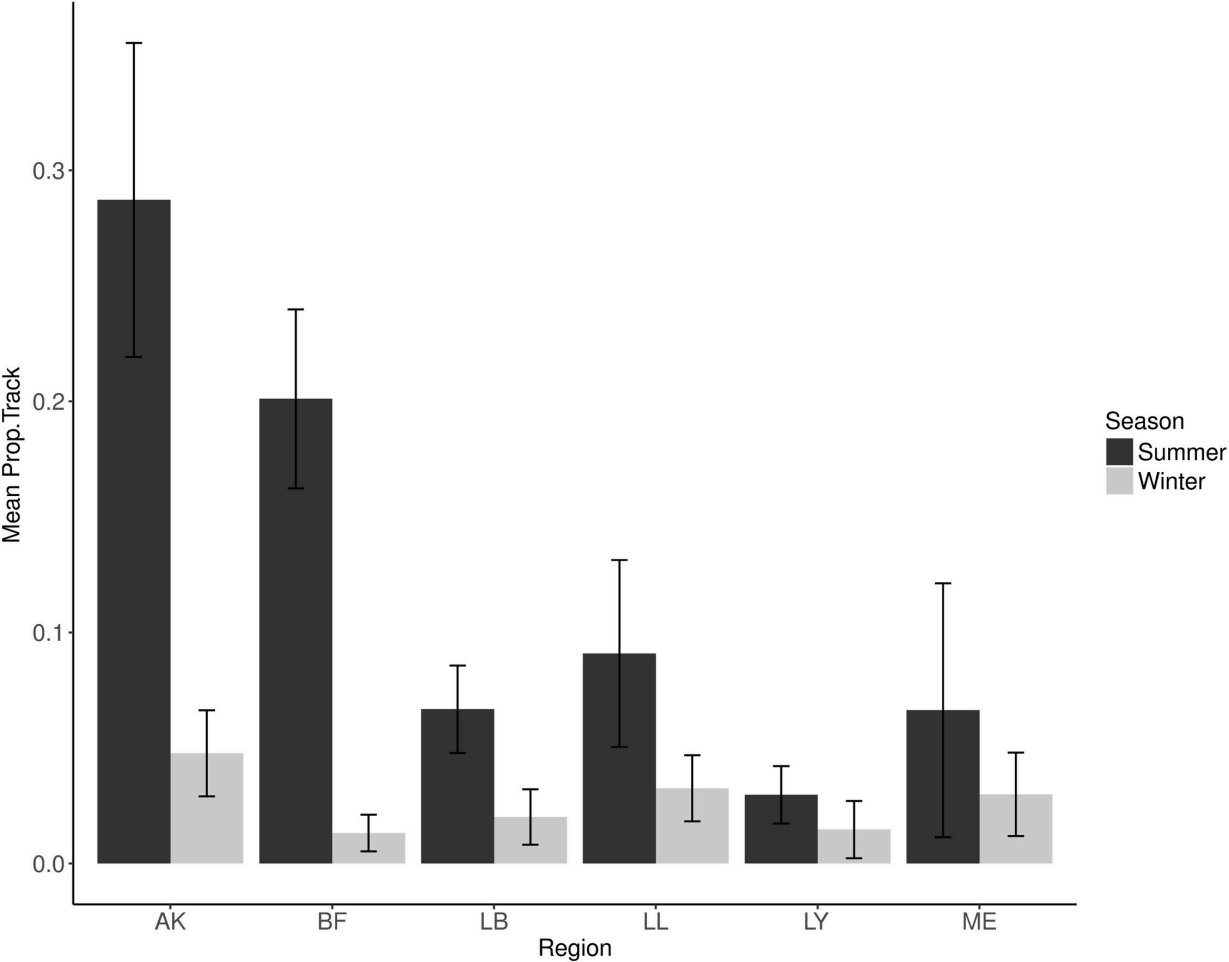
The spatiotemporal distribution of relative abundance of potential prey among survey regions over two seasons. RAPP is summarised here as the proportion of a survey track over which schools are detected (Prop.Track), the best RAPP index for Hector’s dolphins. Error bars are +/- standard error.

There was substantial spatial and temporal variability in the distribution of RAPP. RAPP was higher in summer at all survey regions compared to winter (Fig 7). During summer, RAPP was highest at Akaroa, Birdling’s Flat and Long Lookout, while RAPP was lowest at Lyttelton. In winter, there was little difference in RAPP among survey areas.

Different RAPP metrics were selected as ‘best’ candidates for each predator (Table 3). Prop.Track was the best RAPP metric for predicting the relative abundance of dolphins, while c.SchA was a better predictor of penguin abundance (Table 3). The best GAMMs explained 42.8% and 36.6% of the deviance in the relative abundance of dolphins and penguins, respectively.

**Table 3:** Model selection table.

*Dolphin*	*edf*	*Deviance*	*AIC*
Dolphin ~ Prop.Track + RE(SurveyRegion)	9	42.8%	669
Dolphin ~ c.SchA + RE(SurveyRegion)	8	36.3%	697
***Penguin***			
Penguin ~ c.SchA + RE(SurveyRegion)	8	36.6%	337
Penguin ~ Prop.Track + RE(SurveyRegion)	7	34.6%	339

Model selection table to determine the best RAPP variable for each predator. RE shows the random effects term, edf is the effective degrees of freedom for each GAMM model. Models are ranked by AIC.

**Fig 8:**
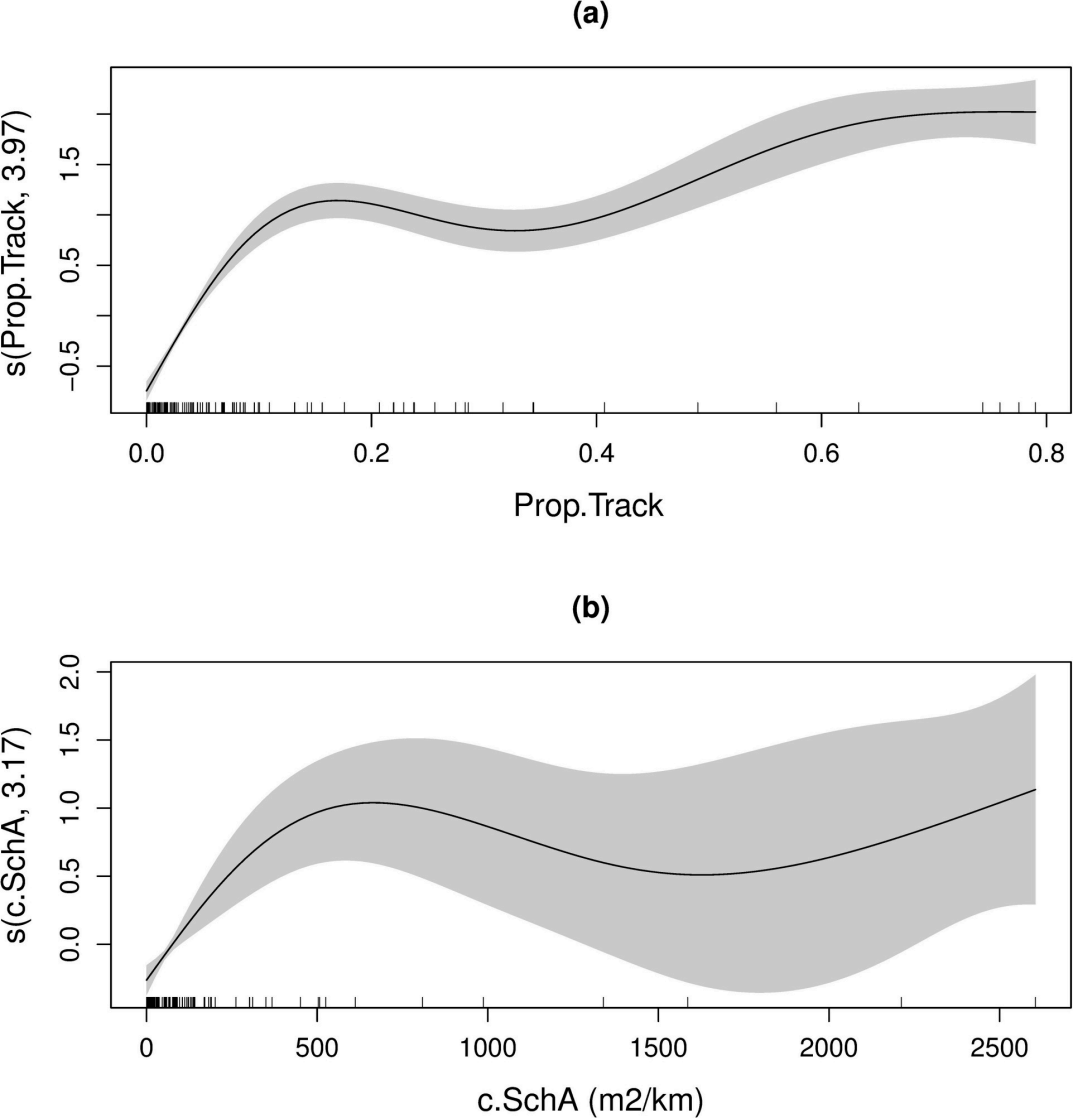
Model plots. The smoothed effects of RAPP on the relative abundance of Hector's dolphins (a) and little penguins (b) from separate GAMMs. Different prey metrics were found to be the best predictor for each predator. The degrees of freedom for each smoothed effect is given on the y-axis. The dashes on the x-axis represent the distribution of the two RAPP metrics. The shaded region is the 95% confidence interval.

RAPP had a strong influence on the relative abundance of both predators (Fig 8). For dolphins, increasing Prop.Track had a positive effect on dolphin counts. The same was true for the penguin model, yet the effect of increasing c.SchA plateaued at approximately 700 m^2^/km, where the effect of c.SchA became less certain (presumably due to a scarcity of observations with very high RAPP). The magnitude of the effects (y-axes; Fig 8) suggest strong overlap between both predators and potential epipelagic prey.

## Discussion

Using recreational grade echosounders, we documented substantial spatial and temporal variability in the relative abundance of potential prey. Schooling fish were more common in our nearshore study area during summer compared to winter, and certain survey regions had markedly high RAPP. Patchiness in space and time is a typical feature of small epipelagic fishes [2,14,17], yet is not well documented in New Zealand, see [20,25,26]. In one of the few NZ studies, Sprat eggs were found at at higher density offshore in the Canterbury Bight in winter/spring seasons; suggesting that the species spawns beyond the nearshore environment at this time of the year [37]. In Australia, pilchards were also found to spawn off the coast (2-8km) between July and December [55] and are more common in shallow, coastal habitat during summer [56]. Hector’s dolphins show a decrease in their use of nearshore habitat during winter [57,58] and are found further offshore at this time [58,59]. This study provides evidence that the dolphin’s seasonal inshore-offshore distribution matches that of their prey.

During summer, the three ‘hotspots’ for Hector’s dolphin had the three highest mean RAPP, suggesting prey plays some role in hotspot formation in this species. Such fine-scale patchiness in RAPP almost certainly reflects the habitat preferences of certain prey species and thus may be influenced by bathymetry, substrate or oceanographic features [17,60,61]. The spatial variability in RAPP seen in this study provides opportunities to examine the factors influencing habitat use by these important mid-trophic level species, information that is currently lacking for New Zealand.

The ground-truthing procedure was useful for determining the school dimensions of potential prey as recorded by our RGE equipment. The distribution of school dimensions is likely to be sensitive to the constraints defined in the SHAPES algorithm, however. Setting smaller school dimensions in SHAPES may have caused problems due to error of GPS fixes from the navigation systems (average error approximately 3m; Navico pers comm.), particularly in the along-track (length) dimension. Further, other backscattering sources (e.g. stochastic artefacts, top predators) may have been included in detected schools (false positives) if smaller dimensions had been set in SHAPES. The frequency distribution of school dimensions showed a peak after the minimum values set by SHAPES, thus it is unlikely that a large number of true schools were missed (false negatives). The distribution of the relative mean school backscatter also showed a peak after the minimum threshold value (-35db). Comparatively few schools had mean backscatter intensity less than -30 dB. Without formal calibration [54], these values cannot be used in any quantitative sense (i.e. to define density). However, the distribution of relative school backscatter intensity provides useful information on relative school density that can assist in the identification of potential prey schools as recorded by these RGEs in shallow coastal habitats. This may be particularly useful when attempting to distinguish echogram marks of different origin (e.g. sediment plumes or zooplankton vs. prey). Knowledge of the relative scattering strength of prey schools also aids in setting appropriate minimum analysis thresholds. As depth is similar throughout the study area, only one suite of settings were used on each echosounder; there was no need to change range settings (and thus source level) or gain, which would influence relative intensity values. Relative intensity values were not used to model predator-prey overlap (e.g. calculating relative scattering area coefficients) due to the underlying uncertainty around the stability of the uncalibrated systems.

The three fish species observed at ground-truthing events provide a good representation of the known epipelagic prey field at Banks Peninsula. All feature in the diet of Hector’s dolphins and/or little penguins [23,30], which were commonly observed at ground-truthing events. That the sample of prey species identified at ground-truthing matches the most important components of predator diet in this area provides confidence that we obtained morphometric and relative intensity data that are representative of known prey. Sprat in particular is a key prey item for both predators, and was the most commonly observed prey species in this study. There was an insufficient number schools with verified identity to examine differences in distribution or school morphology among species. Further studies should establish the identity of a greater range of schools, using different sampling methods (e.g. trawl samples, towed cameras). Such information could be used to define the characteristics of monospecific schools, potentially providing more accurate data to establish predator-prey relationships.

Schools of potential prey were readily detected by the RGEs used in this study. RGEs have also performed well in other studies used to assess the abundance and/or distribution of small schooling fish [17,21,22,62]. Comparisons of a RGE with a Simrad EK60 SES found the systems closely agreed in the estimation of school depth, area, relative abundance and distribution when both sampled the same schools [21]. Similarly, a Humminbird RGE performed well at school detection and classification when run alongside a Biosonics SES [62]. The emerging picture is that RGE systems offer an inexpensive option to provide meaningful data on the distribution and relative abundance of the prey of top predators.

In all echosounder systems (SES or RGE) there is inherent uncertainty in what the echo traces represent. Species identity is seldom certain, though because target strength can be quantified, this is less problematic for SES. While ground-truthing provided valuable data on likely school dimensions and characteristics of known epipelagic prey, other biological aggregations may share these characteristics. This was a particular issue when aggregations of the pelagic phase of squat lobster (*Munida gregaria*) were abundant throughout the study area in the 2016 summer season. *Munida* aggregations were large, dense and had high relative intensity values similar to those of known epipelagic fish schools. Other *Cephalorhynchus* species regularly eat *Munida* in the south of South America [63], and *Munida* is an important component in the diet of many seabirds, including penguins [64,65]. We assume, therefore, that some inclusion of *Munida* aggregations in the assessment of the utility of RGE systems for quantifying potential prey will not strongly influence the relevance of the data for top predators.

Due to the lack of in-situ acoustic calibration and the presentation of backscatter as a generic ‘relative intensity’ rather than Sv format, there is highly likely to be some coarseness to RGE sourced data for linking the distribution of predators to their prey. Reliance on RAPP metrics that are based solely on school dimensions ignores the importance of density, which is normally included in metrics of acoustic relative abundance (e.g. nautical area scattering coefficient; [14,66]). Further, the 3dB transducer beam angle supplied by the manufacturer is likely to be approximate, with the true beam pattern requiring careful calibration [43]. Substantial deviation between the values supplied by the manufacturer and the true beam pattern, will result in error in the estimates of school dimensions. Due to the shallow depths of our study area, it is unlikely that the acoustic beam ensonified schools in their entirety. Thus, it is assumed that the portion of a school detected by the RGE represents a reasonable estimate of the school’s true dimensions, at least in relative terms. Without ground-truthing (e.g. concurrent trawl sampling), there is little scope to test this assumption.

If certain predators prefer particular epipelagic prey (e.g. [14,67]) the inability of the RGE method to identify schools to species level means there will be additional coarseness in unravelling the spatial overlap between trophic levels. Little blue penguins and Hector’s dolphins show inherent flexibility to target the most abundant prey [30,68]. This suggests that metrics summarising the characteristics of a general epipelagic prey field may be appropriate for establishing spatiotemporal concurrence. Such an approach is commonly used to investigate links between the relative abundance of mesopelagic prey and top predators [66,69]

A final limitation is the small volume of water sampled for prey due to the narrowness of the beam in shallow water habitat. This is not a limitation of RGE per se, with SES facing similar challenges in shallow water [70,71]. When prey biomass is low, the chances of a school being ensonified by a small sample volume are greatly reduced [71]. This may result in a negative bias in assessment of relative abundance at times when biomass is low, which would frustrate attempts to extrapolate abundance information to density and biomass estimates [71]. This bias is further accentuated by the avoidance behaviour that fish schools often exhibit towards the survey vessel in shallow water [72] Fish avoidance behavior can result in horizontal displacement [26,43], in which schools are not sampled or only partially sampled by the acoustic beam. Vertical displacement is also common in shallow water and results in bias in the estimation of target depth [43,72]. If depth is variable among survey areas, and avoidance is more common in shallow water, there may be bias in estimation of relative abundance. In this study, depth was relatively consistent across survey areas (between 10 and 35m), yet further work is required to determine whether avoidance behavior affected our estimates of prey relative abundance. It is assumed that the effect of avoidance behaviour on metrics of relative abundance are small, however there is limited opportunity to test this assumption without more extensive sampling to ground-truth measures of relative abundance.

Despite these limitations, there was clearly a strong overlap between predators and potential prey in this study. This provides evidence that RGE can be used as a tool to investigate habitat-use in coastal predators. Distribution models for both predator species explained a good proportion of deviance in the response variable; comparable and in some cases exceeding those from other studies linking predator and prey distribution [5,7,66,69]. Interestingly, each predator had a different ‘best’ RAPP predictor. This could be due to certain prey patch characteristics influencing the detectability and/or exploitability of the prey field for either predator [5]. The 2-dimensional c.SchA metric was more important for penguins compared with the 1-dimensional Prop.Track that was the best predictor of dolphin relative abundance. As visual predators, perhaps school thickness is an important feature of the prey field for penguins that is less relevant to echolocating dolphins. Further research could test the relative importance of RAPP and prey patch characteristics for the two species.

This is the first study to establish overlap between Hector’s dolphins and little penguins with their epipelagic prey. These predators have an endangered and threatened status, respectively [73,74]. Substantial declines in populations of little penguin have been linked to fluctuations in the abundance of epipelagic prey [35,36]. Clearly, understanding the spatiotemporal relationships between these two predators and their prey, and how they may be changing, is important for conservation. The affordability and ease of use of RGE make them useful tools for unravelling such a dynamic and patchy prey field. Such data can be used in species distribution models to appraise the drivers of habitat use in coastal predators; allowing for the identification and subsequent protection of high quality habitat.

Internationally, there has been substantial concern about the state of stocks of small epipelagic fishes [9,29,75]. These taxa are vulnerable to climatic variability [28,75] and overfishing [9,29], and are crucially important to marine ecosystems [76,77]. Despite their importance, and the knowledge of threats facing their populations, little research has focused on the drivers of population dynamics and habitat use. This is especially true in New Zealand, where there have been no published studies on small epipelagic fishes since 1998 [26]. As some regions of New Zealand, including coastal South Island, are showing strong signals of ocean warming [78], such research should take a high priority. While RGEs are already useful, if some of their limitations can be overcome (e.g. cross calibration with SES; [21]), RGE could provide inexpensive, robust tools to investigate the processes underpinning the variability in populations of small epipelagic fishes, and the top predators they support.

## Supporting information

S1 Fig Depth dependence of detected schools. The depth dependence of a mean backscatter values from selection of schools detected during predator-prey surveys is shown before (a) and after (b) correction.
(TIF)

S1 File Datasets used in analyses. The file contains datasets for the ground-truthing exercise and the predator-prey surveys.
(ZIP)
