# Supplementary figures and images for: Using a recreational grade echosounder to quantify the potential prey field of coastal predators
Source: PLoS One. 2019 May 22;14(5):e0217013. doi: 10.1371/journal.pone.0217013 (PMC6530895; doi:10.1371/journal.pone.0217013)

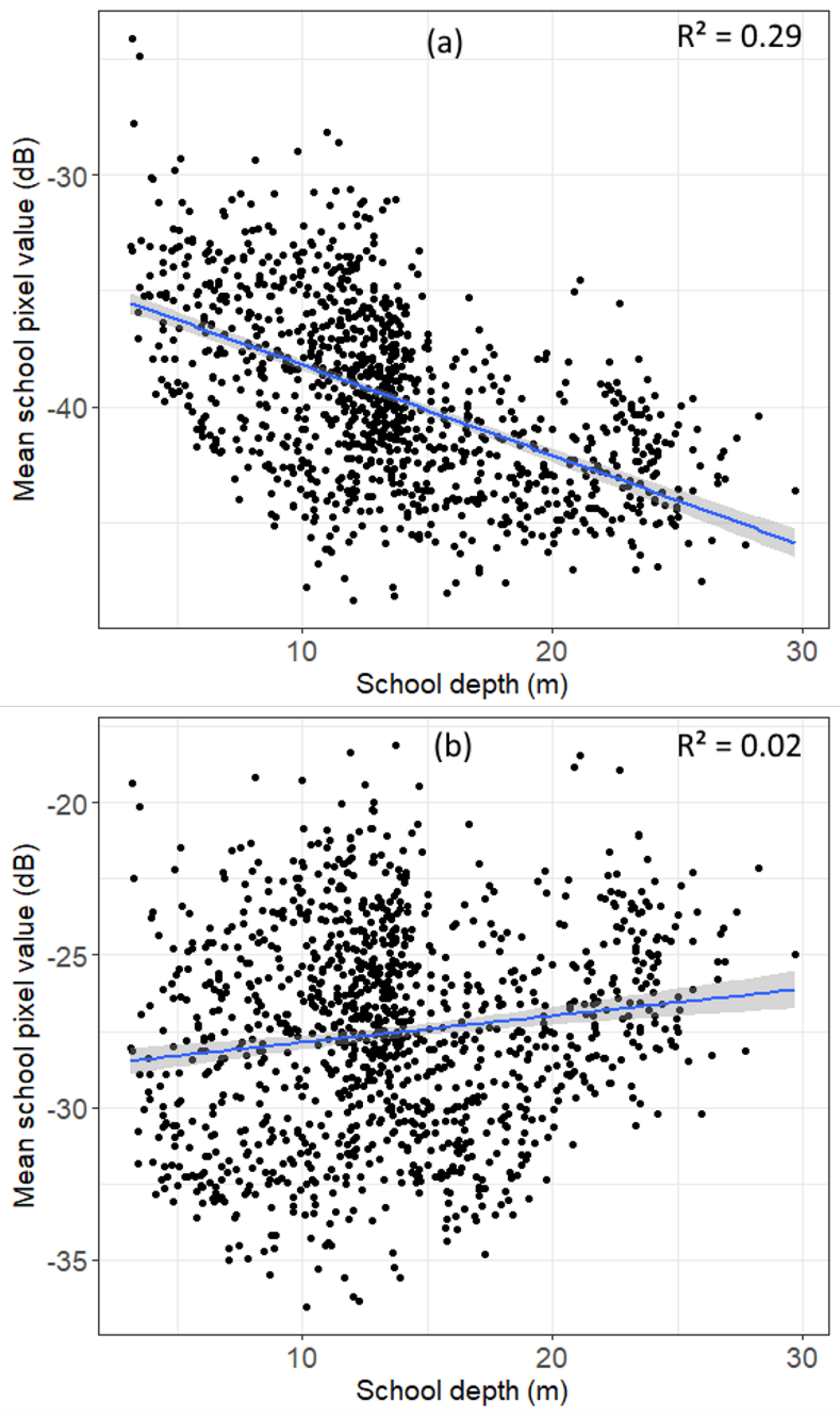

Supplement: S1 Fig — The depth dependence of a mean backscatter values from selection of schools detected during predator-prey surveys is shown before (a) and after (b) correction. (TIF) [file pone.0217013.s001.tif]
